# Motor impairment, balance, and muscle coactivation limit the effectiveness of voluntary corrections of asymmetry during walking after stroke

**DOI:** 10.64898/2026.07.27.26359033

**Published:** 2026-07-29

**Authors:** Andrian Kuch, Samantha Jeffcoat, Alejandro Aguirre-Ramirez, Hailey Hashiguchi, Evan Shrier, Andrew Hooyman, Nicolas Schweighofer, Carolee Winstein, Alison McKenzie, Natalia Sánchez

## Abstract

**Introduction:**

Several gait rehabilitation approaches after stroke rely on explicit feedback to promote task-specific voluntary corrections of walking patterns. While these approaches show effectiveness at a group level, individual responses to voluntary corrections can differ, limiting the benefits and translation of task-specific gait interventions. Our goal is to identify biomechanical, neuromuscular, and cognitive characteristics associated with the ability to perform voluntary corrections of walking using explicit visual feedback in people with chronic stroke.

**Methods:**

Twenty-eight individuals with chronic stroke completed a single-session treadmill walking protocol, consisting of baseline walking, a voluntary correction condition guided by real-time visual feedback, and a short retention trial without feedback. Reducing step length asymmetry was used as the target to guide voluntary corrections. Clinical assessments included measures of motor impairment, balance, gait function, cognition, and walking capacity. Muscle coactivation was characterized using dimensionality reduction. Associations of clinical assessments with baseline step length asymmetry and residual error in step length asymmetry during voluntary correction were examined using univariate analyses and multivariate regression with LASSO-based variable selection.

**Results:**

Eighteen participants successfully reduced step length asymmetry using visual feedback, while ten participants did not reduce asymmetry. Greater residual asymmetry during voluntary correction was independently associated with greater baseline asymmetry, greater lower extremity motor impairment, reduced balance, and increased paretic muscle coactivation (adjusted R² = 0.46). Neither the direction of asymmetry nor cognitive outcome measures were associated with the ability to correct asymmetry during walking. Immediate retention after feedback removal was limited, with only 4 participants maintaining improvements.

**Discussion:**

The ability to perform voluntary corrections of the walking pattern using voluntary corrections after stroke is constrained by motor impairment, balance function, and muscle coactivation. These findings suggest that explicit, feedback-based gait interventions to guide voluntary corrections may benefit individuals with mild to moderate impairments, while individuals with more severe impairments require alternative strategies to guide corrections of the walking pattern.

## Full Text Availability

The license terms selected by the author(s) for this preprint version do not permit archiving in PMC. The full text is available from the preprint server.

